# The Expression Profile and Prognostic Values of EPHA Family Members in Breast Cancer

**DOI:** 10.3389/fonc.2021.619949

**Published:** 2021-06-18

**Authors:** Xixun Zhang

**Affiliations:** Oncology Department, The First Affiliated Hospital of Shantou University Medical College, Shantou, China

**Keywords:** breast cancer, EPHA family, bioinformatics, biomarkers, prognosis

## Abstract

**Background:**

EphAs are a class of ephrin receptors that belong to the membrane-bound receptor tyrosine kinases group. Accumulating experimental evidence has shown that the EphA family is involved in tumor progression, namely in cell proliferation, invasiveness, and metastasis. EphAs are a promising target for anticancer therapy. However, their role in breast cancer (BC) is still not well understood.

**Materials and Methods:**

We used a series of bioinformatic approaches to analyze the expression of the EphA family members and investigate their prognostic value in BC.

**Results:**

Lower expression levels of EphA2, EphA3, EphA4, EphA5, and EphA7 and higher expression levels of EphA10 were found in BC tissues compared to those in normal tissues. The expression levels of the EphA family genes were correlated with molecular subtyping but not with tumor stage. High expression levels of most EphAs indicated a better prognosis in BC.

**Conclusions:**

This study suggested that EphA2, EphA3, EphA4, and EphA5 can act as tumor-inhibiting factors as well as biomarkers for the prognosis of BC.

## Introduction

Each year, worldwide, there are an estimated 1.5 million new cases of breast cancer (BC), which is one of the most frequently diagnosed malignancies in women (1). At present, the strategies for the treatment of BC include surgery, radiation, traditional chemotherapy, endocrine therapy, and targeted therapy. These treatments have greatly improved the progression-free and overall survival of patients with BC. According to the status of ER, PR, and HER2, BCs are divided into four subtypes: luminal A, luminal B, HER2-positive, and basal-like. Once identified, the knowledge on these BC subtypes can be used for clinical decision making to improve the rate of survival for BC. However, the morbidity and mortality associated with BC remain high. The major reason for this is the high recurrence and metastasis rates in patients with BC. Currently, the 5-year survival rate for BC is still less than 20%. Thus, it is necessary to identify reliable biomarkers and new therapeutic targets for BC.

Eph receptors (Ephs) form the largest subfamily of receptor tyrosine kinases (RTKs), with 16 members cloned (2, 3). Based on the types of their binding ligands (ephrins), they can be divided into two subclasses: EphA and EphB. The EphA subfamily has nine members, including EphA1, EphA2, EphA3,EphA4, EphA5, EphA6, EphA7, EphA8, and EphA10. Deregulated activation of the EphA family members has been found in various human cancers, such as lung cancer (4), gastric cancer (5), hepatocellular carcinoma (6), esophageal squamous cell carcinoma (7), and prostate cancer (8). Evidence indicates that EphA receptors are involved in regulating tumor growth, invasiveness, angiogenesis, and metastasis by altering cell proliferation, motility, invasion, and migration (2, 9). For instance, the overexpression of EphA2 contributes to the amplification of ErbB2 signaling, as well as the promotion of BC tumorigenesis and metastasis (10). Overexpression of EphA4 is significantly associated with migration in triple-negative BC (11). MiR-10a interacts with EphA8 and regulates the EMT process (12). Despite these meaningful findings, bioinformatic methods have not been used to explore the expression of the EphA family members in BC.

The EphA family has been identified as a new target for cancer treatment (13, 14). However, it has not been effectively used in BC. Many inhibitor and activator molecules have been designed to target the EphA family members (15–17). Some of them work by inhibiting the kinases of the EphAs, while some others work by inhibiting the expression of the EphA family members (18–20). However, our limited understanding of EphA activity and the EphA expression pattern in cancers represents a challenge in the application of therapeutic strategies. Thus, a thorough understanding of their involvement in BC is needed.

Bioinformatics provides an effective and feasible method for the analysis of tumors and has become an essential component of biomedical studies. The advantage of using bioinformatics is its ability to explore and collect various data from numerous studies in an unbiased way, which can help us obtain useful information about cancer progression. In this study, we investigated the expression profiles, mutation status, and prognostic values of the EphA family members using bioinformatics and identified prognostic factors for BC.

## Materials and Methods

### Oncomine Database Analysis

The Oncomine database (https://www.oncomine.org/resource/login.html) was used in this study to analyze the expression levels of the EphA family genes in different types of cancers. The p-value was defined as 0.01, and the fold change was defined as 2.

### GEPIA Data Set Analysis

GEPIA is an online database consisting of 9,736 tumor and 8,587 normal control samples (http://gepia.cancer-pku.cn/index.html). The GEPIA database was used in this study to analyze the transcription levels of the EphA family members in BC. Student’s *t* test was used to produce the p value. The p value cutoff was 0.05.

### UALCAN Database Analysis

UALCAN is a database that can analyze cancer OMICS data (http://ualcan.path.uab.edu/). In this study, the UALCAN database was used to analyze the correlation between EphA family member expression and molecular subtyping in patients with BC. Student’s t test was used to produce the p value. The p value cutoff was 0.05.

### Kaplan-Meier Plotter Analysis

The Kaplan-Meier plotter (http://kmplot.com/analysis/) consists of a variety of genes in different types of cancers, such as breast (n = 6,234), ovarian (n = 2,190), lung (n = 3,452), and gastric (n = 1,440) cancer cohorts. In this study, the Kaplan-Meier plotter was used to analyze the prognostic significance of EphA family gene expression in patients with BC.

### cBioPortal Database Analysis

The cBioPortal (http://www.cbioportal.org/) is attached to the Memorial Sloan Kettering Cancer Center for Cancer Genomics, and it can provide a comprehensive analysis of complex tumor genomics and clinical profiles in The Cancer Genome Atlas (TCGA). Using the cBioPortal database, we investigated the frequency and form of EphA gene mutations.

### COSMIC Database Analysis

The COSMIC database (http://cancer.sanger.ac.uk) is a high-resolution resource for studying the effects of somatic mutations in all types of human tumors. In this study, the COSMIC database was used to analyze mutations in the EphA family in BC. Pie graphs were used to depict the substitutions of the EphA genes.

### STRING

STRING (https://string-db.org/) is a database that collected all publicly available sources of protein–protein interaction. In this study, we conducted a protein–protein interaction network analysis of EphAs to explore the interactions among them with STRING.

### GeneMANIA Database Analysis

GeneMANIA (http://www.genemania.org) is a website that provides information on protein and genetic interactions, pathways, co-expression, co-localization, and protein domain similarity of the submitted genes.

### WebGestalt

WebGestalt (http://www.webgestalt.org/) is a functional enrichment analysis web tool. In this study, we explored the potential function of EphAs with WebGestalt. FDR Method is BH.

## Results

### EphA2, EphA3, EphA4, EphA5, and EphA7 Expression Is Lower, and EphA10 Expression Is Higher in BC Tissues

**Figure 1:**
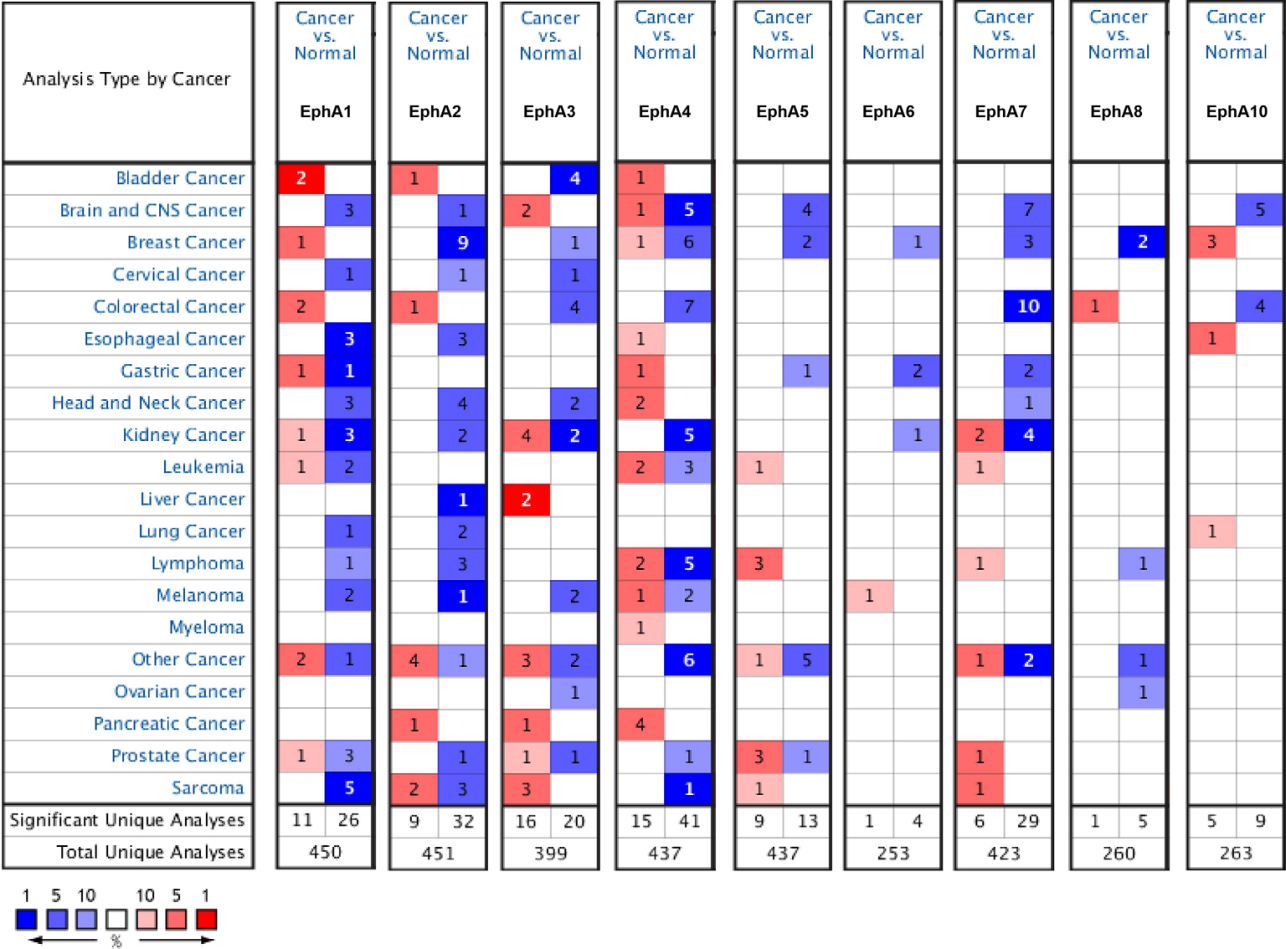
The mRNA expression pattern of EphA family members in different tumor types. The graphs generated from the Oncomine database show the numbers of data sets with statistically significant expression of EphA family in different types of tumor tissues and normal tissues. High expressions were showed in red block charts, and low expressions were shown in blue block charts. The threshold was defined with the following parameters: *P*-value of 0.01, fold change of 2 and gene ranking of top 10%. Cell color is determined by the best gene rank percentile for the analyses within the cell.

**Figure 2:**
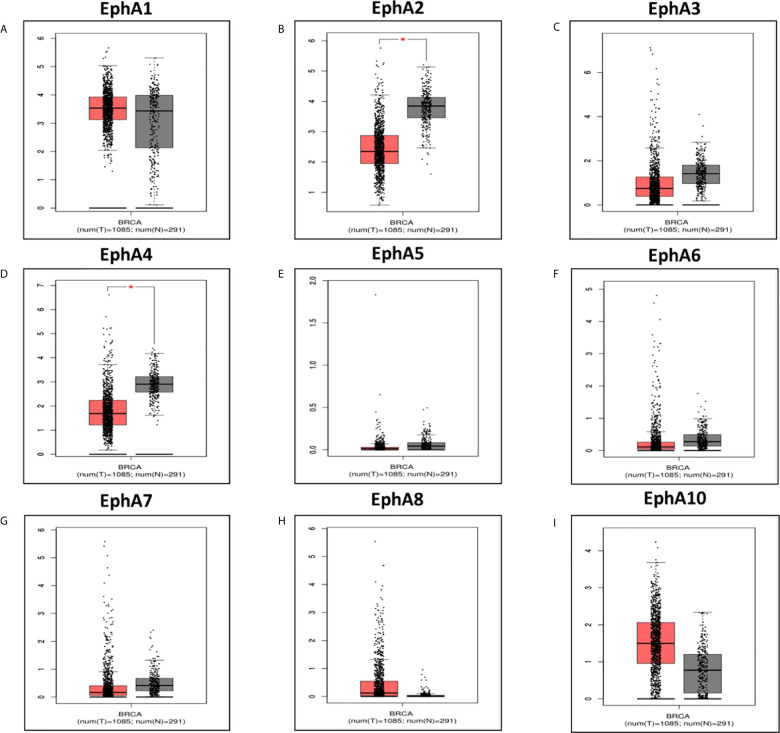
The expression of EphA family in breast cancer vs in normal. The graphs generated from GEPIA database showed the EphA family gene expression in breast cancer tissues (BRCA) (n = 1085) compared with normal breast tissues (n = 291), *P < 0.05. The tumor tissues were shown in red and the normal tissues were shown in gray. The EphA family genes include **(A)** EphA1; **(B)** EphA2; **(C)** EphA3; **(D)** EphA4; **(E)** EphA5; **(F)** EphA6; **(G)** EphA7; **(H)** EphA8; **(I)** EphA10.

Eight EphA factors have been identified in humans. The expression levels of EphAs were compared between the cancer samples and normal samples using the Oncomine database (**Figure 1**). The results showed that the expression levels of EphA2, EphA3, EphA4, EphA5, EphA7, and EphA8 were reduced, but the expression levels of EphA1 and EphA10 were significantly increased in BC tissues (P < 0.01). To further confirm these results, the expression levels of the EphA family members were compared between the BC and normal tissues using the GEPIA database **Figure 2**. The results indicated that the expression levels of EphA2, EphA3, EphA4, EphA5, EphA6, and EphA7 were significantly lower in BC tissues than in normal tissues, and the expression levels of EphA8 and EphA10 were significantly higher in BC tissues. Consequently, we concluded that EphA2, EphA3, EphA4, EphA5, and EphA7 were downregulated, while EphA10 was upregulated in BC tissues.

### The Expression of EphA Family Members Is Typically Correlated With Molecular Subtyping But Not Tumor Stage

**Figure 3:**
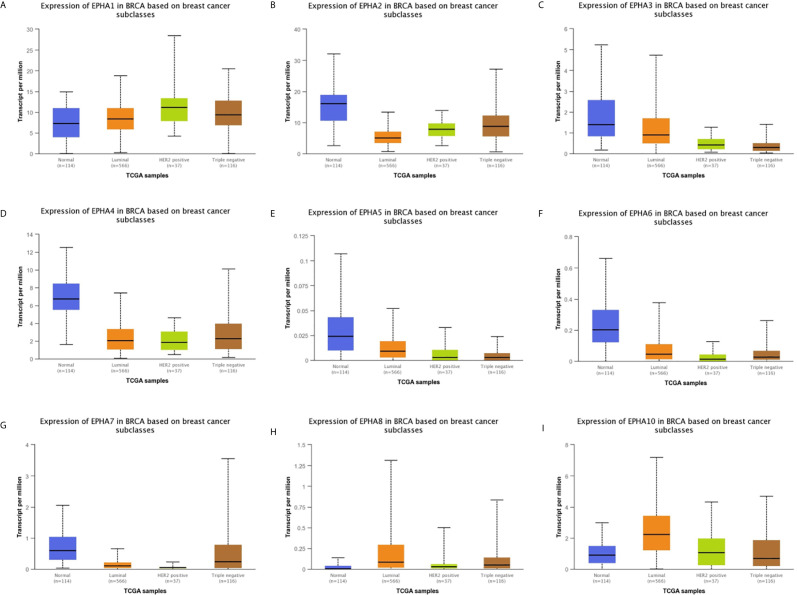
The correlation between EphA family expression and molecular subtyping in breast cancer patients. The graphs produced from the UALCAN database showed the expression of EphA family genes in different molecular subtyping of breast cancer. The normal tissues were shown in red, the liminal types were shown in orange, the Her2-positive type were shown in green and the triple negative type were showed in brown. The EphA family genes include **(A)** EphA1; **(B)** EphA2; **(C)** EphA3; **(D)** EphA4; **(E)** EphA5; **(F)** EphA6; **(G)** EphA7; **(H)** EphA8; **(I)** EphA10.

**Figure 4:**
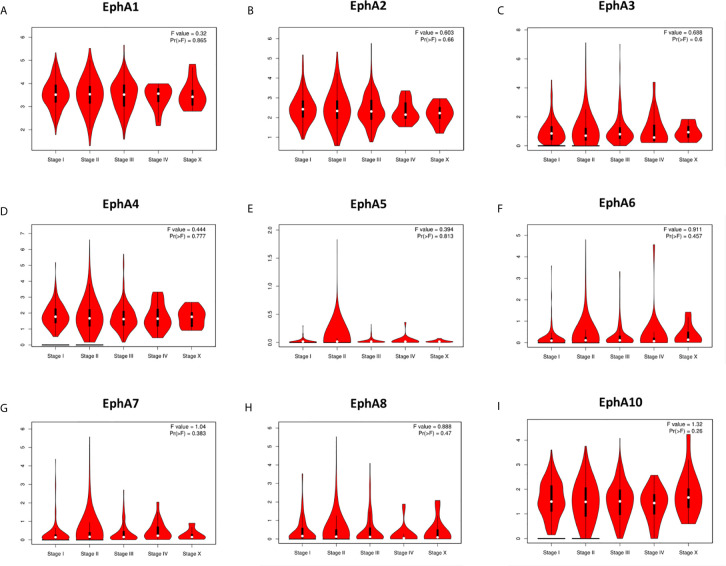
The correlation between EphA family expression and tumor stage of breast cancer patients. The graphs generated from the GEPIA database showed the expression of EphA family genes in different tumor stages of breast cancer. The EphA family genes include **(A)** EphA1; **(B)** EphA2; **(C)** EphA3; **(D)** EphA4; **(E)** EphA5; **(F)** EphA6; **(G)** EphA7; **(H)** EphA8; **(I)** EphA10.

We analyzed the correlation between the expression of the EphA family members and molecular subtyping in patients with BC **Figure 3**. We found that most EphA groups varied significantly. EphA1 was significantly high in HER2-positive BC; EphA2 and EphA7 were significantly high in triple-negative BC, and EphA3, EphA5, EphA6, EphA8, and EphA10 were significantly high in luminal BC. The EphA4 groups did not show any significant differences. We further analyzed the relationship between EphA family member expression and tumor stage in patients with BC using the GEPIA database. **Figure 4** The results showed that none of the EphA members differed significantly.

### High Expression of Most EphAs Predicts Better Prognosis in BC

**Figure 5:**
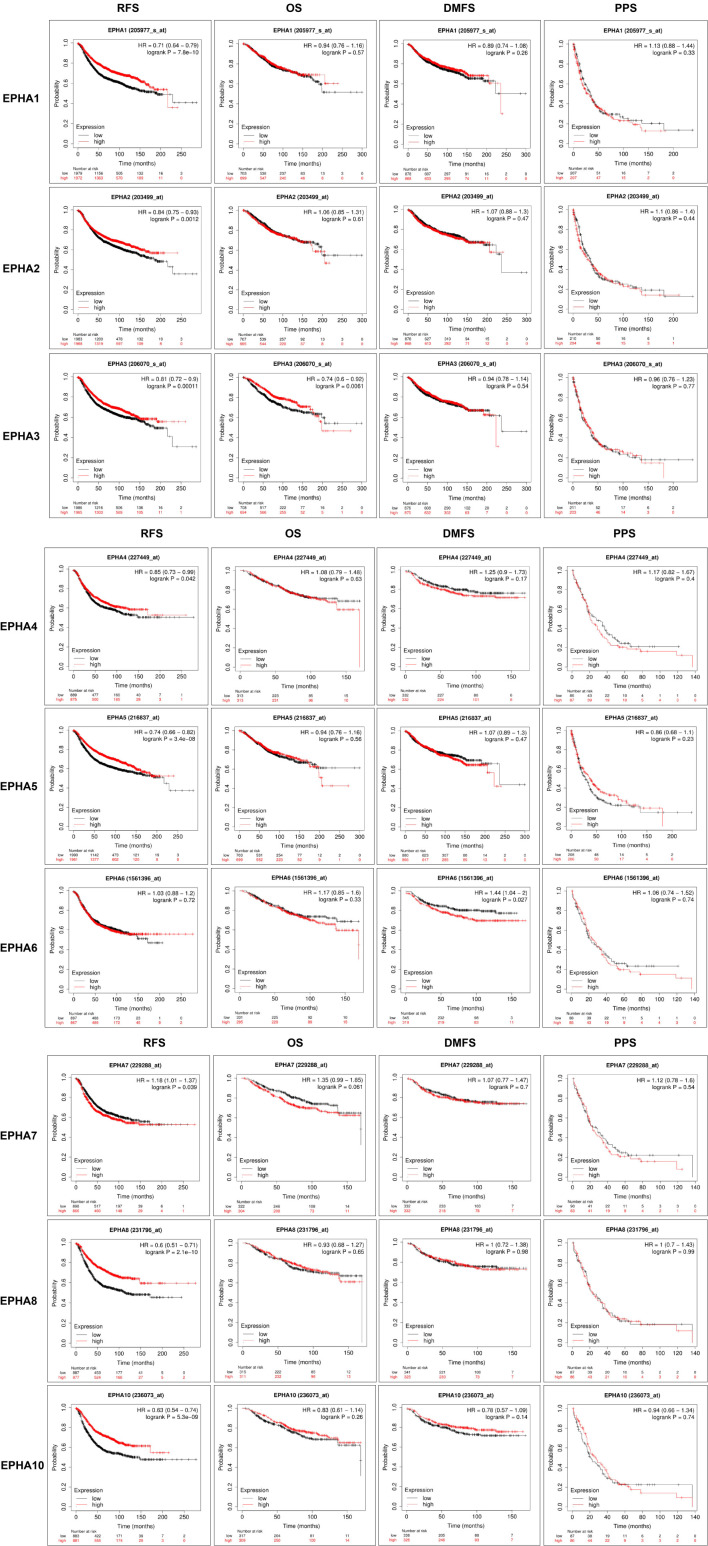
The prognostic significances of EphA family genes expression in breast cancer patients. The curves generated from the KM plotter database showed the prognostic value of EphA family genes. The assessments include RFS, OS, DMFS, and PPS. The high expression was showed in red curves and the low expression was showed in black curves. The log rank test was displayed at the upper right corner of every graph.

To explore the prognostic values of EphAs in patients with BC, the Kaplan-Meier Plotter was used to analyze the correlation between the expression of EphAs and survival of patients with BC **Figure 5**. The Kaplan-Meier curve and log rank test analyses revealed that the increased EphA1, EphA2, EphA3, EphA4, EphA5, EphA8, and EphA10 mRNA levels and decreased EphA7 mRNA levels were significantly associated with recurrence-free survival (RFS) (P < 0.05) in all patients with BC. Furthermore, the increase in EphA3 expression was significantly associated with overall survival (P < 0.05), and the decrease in EphA6 expression was significantly associated with distant metastasis-free survival (DMFS) (P < 0.05).

### EphA3, EphA5, and EphA7 Gene Mutations Are Frequent in BC

**Figure 6:**
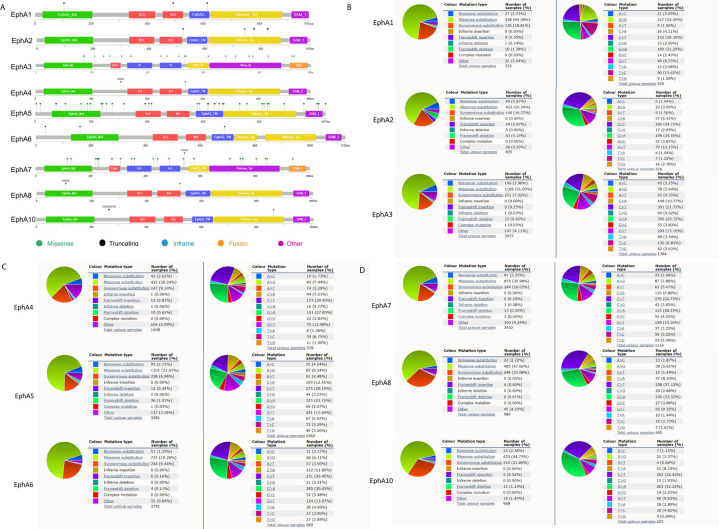
The mutation frequency and mutation types of EphA family genes in breast cancer patients. **(A)** The schematic diagrams produced from the cBioportal database showed the mutation frequency of EphA family through. **(B)** The pie graph produced from the Catalogue of Somatic Mutations in Cancer database showed the percentages of mutation types of EphA family.

Gene mutations may be an important form of low EphA expression. We evaluated the frequency changes of EphA mutations in BC samples using the cBioPortal database. The mutation frequencies of EphA1, EphA2, EphA4, and EphA6 were quite low, only 0.1% (**Figure 6A**). The mutation frequencies of EphA8 and EphA10 were even lower (less than 0.1%) (**Figure 6A**). The mutation frequencies of EphA3, EphA5, and EphA7 were high (0.5%, 0.6%, and 0.4%, respectively) (**Figure 6A**). Furthermore, we analyzed the mutation types of the EphA family members using the COSMIC database. The pie graph showed that the common mutation types in the EphA family members included missense substitutions, synonymous substitutions, and nonsense substitutions. The most common mutation types in EphA1, EphA2, EphA3, EphA4, EphA5, EphA8, and EphA10 are missense substitutions (**Figures 6B–D**).

### Co-expression, Interaction, and Functional Analyses of EphA Family Members

**Figure 7:**
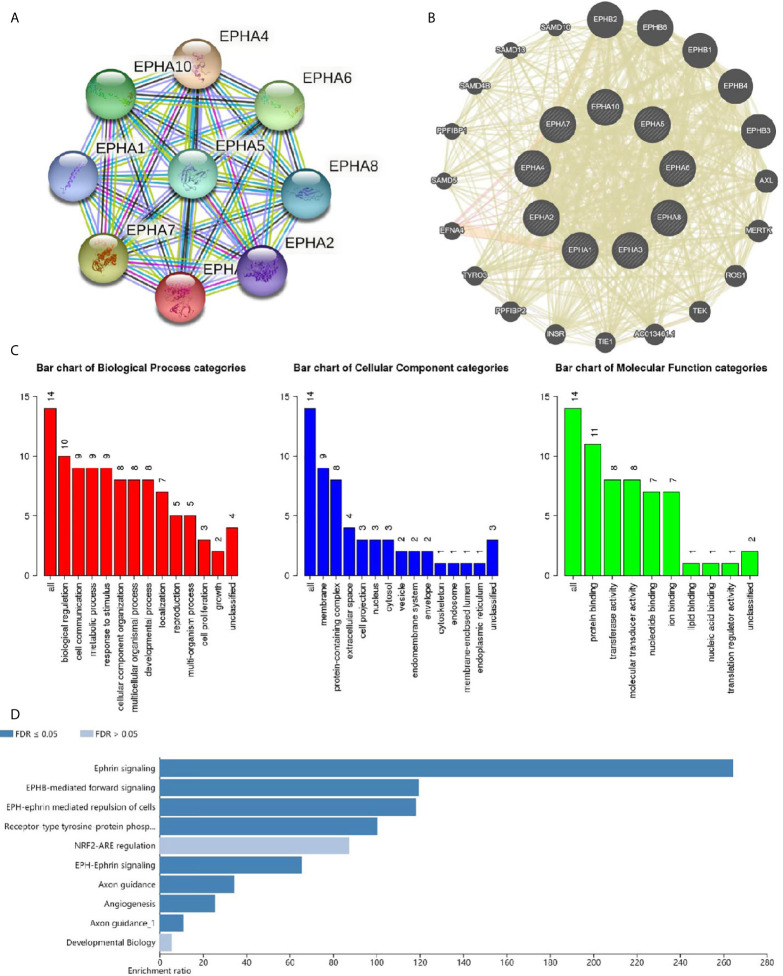
Co-expression and functional analyses for EphA family. **(A)** Protein-protein interaction network of EphA family. **(B)** Protein-protein interaction network analyses for EphA2, EphA3, EphA4, and EphA5. **(C)** Bar plot of GO enrichment in cellular component terms, biological process terms, and molecular function terms for genes in **(B)** .**(D)** Bar plot of KEGG enriched terms for genes in **(B)**.

Next, we explored the potential co-expression and function of the EphA family. We conducted a protein-protein interaction network to analyze the EphAs using STRING to explore the potential interactions among them. As expected, every of EphAs co-expressed with each other tightly (**Figure 7A**). Next, we explored the potential interactions partners with the key genes (EphA2, EphA3, EphA4, and EphA5). GeneMANIA results revealed that EFNA5, EFNA4, EFNA1, EFNA3, EFNA2, ARHGEF15, EFNB2, ACP1, NGEF, CHN1, ADAM10, EFNB1, EFNB3, BLK, MAP3K1, CDK5, FGR, YES1, EPHB2, and HCK were primarily associated with the modulation and function of EphA2, EphA3, EphA4, and EphA5 (**Figure 7B**). GO analyses were performed using WebGestalt. Among the most highly enriched functions in the biological process category, biological regulation, cell communication, metabolic process, response to stimulus, cellular component organization, multicellular organismal process, developmental process, localization reproduction, multi-organism process, cell proliferation, and growth were associated with the expression of EphA2, EphA3, EphA4, EphA5, and their interactions partners. The membrane, protein-containing complex, extracellular space, cell projection, nucleus, cytosol, vesicle, endomembrane system, envelope, cytoskeleton, endosome, membrane-enclosed lumen, and endoplasmic reticulum were enriched in the cellular component category. In the molecular function categories, EphA2, EphA3, EphA4, EphA5, and their co-expression genes were mainly enriched in protein binding, transferase activity, molecular binding, ion binding, lipid binding, nucleic binding, and translation regulator activity (**Figure 7C**). KEGG pathway analyses were also performed with these interactions partners. The result showed that these genes were involved in several pathways, such as Axon guidance and MAPK signaling (**Figure 7D**).

## Discussion

The high expression of EphAs in almost all types of solid tumors indicates aggressive phenotypes and poor prognosis in BC. However, our results seem to show the opposite. Based on our results, we concluded that EphA2, EphA3, EphA4, and EphA5 were down-regulated in BCs, and high expression levels of these genes indicated better RFS, suggesting that EphA2, EphA3, EphA4, and EphA5 act as tumor suppressors in BC and could be new biomarkers for its prognosis.

EphA2 is the most widely studied gene in the EphA family. Aberrant expression of EphA2 has been associated with many human malignancies, such as lung cancer (4), breast cancer (21), ovary cancer (22), esophageal cancer (7), colorectal cancer (23), glioblastoma (24), and melanoma (25). However, its function remains unclear. Overexpression of EphA2 has been reported to inhibit cancer cell proliferation and motility, indicating that EphA2 can act as a tumor suppressor (26). The expression level of EphA2 is elevated in malignant mammary glands, indicating that EphA2 may be an oncogenic factor (27). In our study, we found that the expression level of EphA2 was reduced in BC, and up-regulation of EphA2 predicted better RFS, indicating that for this type of tumor, EphA2 can act as a tumor-inhibiting factor.

High expression levels of EphA3 are associated with poor prognosis in gastric cancer (28), colorectal cancer (29), and hepatocellular carcinoma (30). In our study, we found that EphA3 expression was low in BC, and a high expression level of EphA3 predicted a better prognosis.

EphA4 is an essential factor for TGFβ-induced migration associated with later tumor stages, worse prognosis, and chemotherapy resistance, and can be regulated by TGFβ (11). In our study, EphA4 expression was low in BC, and high expression of EphA4 predicted better RFS.

In previous studies, the methylation of EphA5 was associated with later tumor stages and progesterone receptor-negative status in BC (31). However, the results of this study showed the opposite. Our data showed that EphA5 was significantly overexpressed in patients with luminal BC, and there was no correlation between EphA5 expression levels and tumor stage in patients with BC.

The data for EphA1 and EphA10 showed something meaningful. The expression level of EphA1 and its association with clinical parameters are factors that have been analyzed for various tumors, including gastric cancer, colorectal cancer, non-melanoma skin cancer, and squamous cell carcinoma (32–35). EphA1 is a regulator of ERα transcriptional activity in BC cells (36). In this study, we found that EphA1 was highly expressed in HER2-positive BC. It has also been reported that EphA10 is a promising drug target that is potentially useful for BC treatment. EphA10 is down-regulated in triple-negative BCs, and an anti-EphA10 monoclonal antibody can suppress tumor growth (37). The expression of EphA10 in other subtypes is not yet clear. In this study, we found that EphA10 was overexpressed in BC, especially in luminal BC patients, indicating that EphA10 may be a useful therapeutic target for patients with luminal BC.

Mutations in some Eph receptors are predicted to impair kinase function. Evidence shows that targeted null mutation of EphA4 causes defects in the anterior commissure (38, 39), indicating that mutation inactivates the function of EphAs. In our study, we found that mutations in EphA3, EphA5, and EphA7 are frequent, and their expression is low in BC. Thus, we believe that mutations are an important cause of EphA3, EphA5, and EphA7 inactivation. However, the clinical value of EphA mutations requires further study.

## Data Availability Statement

The datasets analyzed for this study can be found in the Oncomine database, GEPIA database, UALCAN database, Kaplan-Meier Plotter database, cBioPortal database, COSMIC database, String, GeneMANIA database and WebGestalt database.

## Author Contributions

XZ designed the study, analyzed the data, edited the figure, wrote, and submitted the manuscript. All authors contributed to the article and approved the submitted version.

## Funding

This study was supported by the Youth Program of National Natural Science Foundation of China (81802956).

## Conflict of Interest

The author declares that the research was conducted in the absence of any commercial or financial relationships that could be construed as a potential conflict of interest.
